# Compared Heritability of Chronotype Instruments in a Single Population Sample

**DOI:** 10.1177/07487304211030420

**Published:** 2021-07-27

**Authors:** Mario A. Leocadio-Miguel, Francieli S. Ruiz, Sabrina S. Ahmed, Tâmara P. Taporoski, Andréa R. V. R. Horimoto, Felipe Beijamini, Mario Pedrazzoli, Kristen L. Knutson, Alexandre C. Pereira, Malcolm von Schantz

**Affiliations:** * Department of Physiology and Behavior, Federal University of Rio Grande do Norte, Natal, Brazil; † Faculty of Health and Medical Sciences, University of Surrey, Guildford, UK; ‡ Northwestern University Feinberg School of Medicine, Chicago, Illinois, USA; § InCor, University of São Paulo School of Medicine, São Paulo, Brazil; || Department of Biostatistics, School of Public Health, University of Washington, Seattle, Washington, USA; ¶ Federal University of Fronteira Sul, Realeza, Brazil; # School of Arts, Sciences, and Humanities, University of São Paulo, São Paulo, Brazil

**Keywords:** circadian rhythms, cohort study, complex traits, diurnal preference, genetic variance

## Abstract

It is well established that the oldest chronotype questionnaire, the morningness-eveningness questionnaire (MEQ), has significant heritability, and several associations have been reported between MEQ score and polymorphisms in candidate clock genes, a number of them reproducibly across populations. By contrast, there are no reports of heritability and genetic associations for the Munich chronotype questionnaire (MCTQ). Recent genome-wide association studies (GWAS) from large cohorts have reported multiple associations with chronotype as assessed by a single self-evaluation question. We have taken advantage of the availability of data from all these instruments from a single sample of 597 participants from the Brazilian Baependi Heart Study. The family-based design of the cohort allowed us to calculate the heritability (h^2^) for these measures. Heritability values for the best-fitted models were 0.37 for MEQ, 0.32 for MCTQ, and 0.28 for single-question chronotype (MEQ Question 19). We also calculated the heritability for the two major factors recently derived from MEQ, “Dissipation of sleep pressure” (0.32) and “Build-up of sleep pressure” (0.28). This first heritability comparison of the major chronotype instruments in current use provides the first quantification of the genetic component of MCTQ score, supporting its future use in genetic analysis. Our findings also suggest that the single chronotype question that has been used for large GWAS analyses captures a larger proportion of the dimensions of chronotype than previously thought.

In human chronobiology, questionnaire-based chronotype is used as a simple proxy of how the circadian period (*tau*) interacts with zeitgebers, thus influencing an individual’s phase of entrainment. Through the interaction between an individual’s length of *tau* and external factors, the phase of entrainment may differ (Duffy and Czeisler, 2002), giving rise to the broad spectrum of chronotypes, ranging from extreme morning types to extreme evening types. The assessment of chronotype is based on questionnaires designed to access the preferred timing of physical and cognitive efforts, as well as sleep-wake timing, as is the case of the morningness-eveningness questionnaire (MEQ) (Horne and Östberg, 1976). The endogenous nature of MEQ has been corroborated by controlled constant routine experiments (Duffy et al., 1999; Kerkhof and Van Dongen, 1996). The dimensions of MEQ have recently been further explored through an exploratory factor analysis (EFA) which reported that MEQ measures three correlated constructs: “Efficiency of the dissipation of sleep pressure,” “Sensitivity to the build-up of sleep pressure,” and a more inconsistent third factor, “Peak time of cognitive arousal” (Panjeh et al., 2021), making it suitable to infer the mutual influences of the circadian and the homeostatic processes (Borbély, 1982). An alternative way to explore chronotype is by assessing an actual entrainment phase (e.g., the half-way point between sleep onset and sleep offset), as used in the Munich chronotype questionnaire (MCTQ) (Roenneberg et al., 2003). Although the MCTQ is simpler and faster to administer than the MEQ, time and space are at a premium in comprehensive questionnaires administered to large cohorts. This was the case for the UK Biobank Project, in which the enrolment questionnaire administered to more than half a million participants was designed to gather data on multiple aspects of health and well-being in approximately 1 h (Ollier et al., 2005). In the UK Biobank, chronotype was assessed through the answer to a single question that closely resembles the last question of the MEQ (“One hears about ‘morning’ and ‘evening’ types of people. Which one of these types do you consider yourself to be?”). The commercial DNA-testing company 23andMe collected chronotype data using an even simpler version, including only the options “night owl,” “early bird,” and “neither” (Hu et al., 2016).

Heritability is the quantification of the overall phenotypic variation that is attributable to genetic factors. It allows the comparison of the relative importance of genes to the variation of traits, such as the circadian preference phenotype, within and across populations (Mayhew and Meyre, 2017; Visscher et al., 2008). No measures are available for the heritability of human *tau*, although it has been calculated to be 0.61 in founder populations of inbred mice and 0.33 in diversity outbred offspring (Keenan et al., 2020). Different measures of chronotype have been used for estimating heritability both in twin and family studies (see Table 1 for a systematic summary), ranging between 0.21 and 0.54, depending on instrument and population. Published association studies between polymorphisms in candidate circadian clock genes and chronotype have focused on the MEQ scale. Several such associations have been reported and, in a number of instances, reproduced (reviewed in von Schantz, 2017). The development of genome-wide association studies (GWAS) enables the hypothesis-free search for genetic associations of phenotypic traits. No significant associations for chronotype or diurnal preferences have been reported from GWAS using MEQ or MCTQ. However, the use of single questions, as described above, in large cohorts has been successful. A combined meta-analysis including chronotype assessed through a single question in both the UK Biobank cohort and in the 23andMe user survey (*n* = 697,828) reported associations with 351 loci (Jones et al., 2019). Heritability for single-question chronotype, as estimated through GWAS, has varied between 12% and 21% (Hu et al., 2016; Jones et al., 2016, 2019; Lane et al., 2016).

**Table 1.** Heritability (h^2^) estimates for circadian preference in twin and family studies.

Study	Method of Assessing Heritability	Chronotype Measure	h^2^ (SE, 95% CI)
Twin studies			
Toomey et al. (2015)	**Additive genetic influences**: maximum likelihood–based structural equation modeling package OpenMx, using the Cholesky analysis.	MEQ score	0.42 (0.34, 0.50)
Barclay et al. (2014)	**Additive genetic influences**: structural equation modeling using the Mx package.	rMEQ (reduced 5-item MEQ)	0.52 (0.46, 0.57)
Watson et al. (2012)	**Additive genetic influences**: structural equation modeling using the Cholesky analysis.	rMEQ (reduced 5-item MEQ)	0.40 (0.27, 0.47)
Barclay et al. (2010)	**Additive genetic influences:** maximum likelihood–based structural equation modeling using the Mx package.	MEQ score	0.52 (0.20, 0.61)
Koskenvuo et al. (2007)	**Additive genetic influences:** maximum likelihood–based structural equation modeling using the Mx package.	Single question according to the diurnal type scale: “Will you try to estimate to what extent your being a morning or an evening people?” [*sic*]	0.49 (0.46, 0.52)
Hur (2007)	**Additive genetic influences:** maximum likelihood–based structural equation modeling using the Mx package.	CS: adapted 13-item MEQ	0.45 (0.39, 0.50)
Vink et al. (2001	**Additive genetic influences:** structural equation modeling using the Mx package.	Final ME question: “Are you a morning-active person or an evening-active person?”	0.44 for adolescents and 0.47 for older individuals in their mid-40s
Hur et al. (1998)	**Additive genetic influences:** maximum likelihood–based modeling using the Mx package.	Abbreviated 13-item MEQ	0.54 (0.45, 0.62)
Family studies			
von Schantz et al. (2015)	**Additive genetic influences:** polygenic heritability estimates kinship2 package.	MEQ	0.48 ± 0.08*
Evans et al. (2011)	**Additive genetic influences:** linear multivariate regression with variance component analysis using software SOLAR.	MEQ	0.21 ± 0.09*
Klei et al. (2005)	**Additive genetic influences:** package Solar2	CS: adapted 13-item MEQ	0.237 ± 0.09*
GWAS studies			
Jones et al. (2019)	SNP-based heritability assessed by BOLT-REM (Loh et al., 2015): **additive genetic influences** of chronotype calculated in the UK Biobank alone.	Single question (MEQ Question 19)	0.137 (0.13, 0.14)
Jones et al. (2016)	SNP-based heritability assessed by LD score regression (Bulik-Sullivan et al., 2015): **additive genetic influences** of chronotype calculated in the UK Biobank alone.	Single question: (MEQ Question 19)	0.12 (NA)
Lane et al. (2016)	SNP-based heritability assessed by LD score regression (Bulik-Sullivan et al., 2015): **additive genetic influences** of chronotype.	Single question: “Do you consider yourself to be?” with possible answer “Morning/evening person (chronotype)”	0.194 (NA)
Hu et al. (2016)	SNP-based heritability assessed by genome-wide complex trait analysis (Yang et al., 2011): **additive genetic influences** of chronotype.	Combination of 2 questions: “Q1: Are you naturally a night person or a morning person? (Night owl, Early bird, Neither)” and MEQ Question 19	0.21 (0.13, 0.29)

Abbreviations: CI = confidence interval; SE = standard error; MEQ = morningness-eveningness questionnaire; SOLAR = Sequential Oligogenic Linkage Analysis Routines; GWAS = genome-wide association studies; SNP = single-nucleotide polymorphism.

*Note*: *p < 0.05

Despite the growing amount of knowledge on the heritability of diurnal preference assessed with the MEQ, to the best of our knowledge, there is no published study of the heritability of chronotype as estimated using MCTQ. The phase of entrainment as estimated by the MCTQ has been reported to show a higher correlation with the MEQ score on free days than on workdays (Zavada et al., 2005; Roenneberg et al., 2007; Miguel et al., 2014). This probably reflects the fact that the MCTQ describes an overt behavioral marker of chronotype rather than a psychological construct of circadian preference (Di Milia et al., 2013). Therefore, it would be plausible to ask to what extent it displays any heritability.

We have previously used data from the Baependi Heart Study cohort to estimate the heritability of a number of phenotypes (Egan et al., 2016; Taporoski et al., 2019). The cohort is a family-based cohort study located in a small rural town in South-Eastern Brazil with little inbound migration and a cohesive culture and lifestyle and has therefore proven useful for estimating the heritability of behavioral measures. We took advantage of the availability of both MEQ and MCTQ data from a sample of 597 participants in this study to calculate the heritability of both, as well as of the single chronotype question, and the factors of the MEQ described above, in the same group of individuals. The protocol for this study conformed to international ethics standards based on the Declaration of Helsinki and was approved by the local ethics committee (Hospital das Clínicas, University of São Paulo, Brazil, number 0494/10). Each volunteer provided informed consent before participation. The inclusion criteria for the present analysis include having completed both chronotype questionnaires (MEQ and MCTQ). MEQ data were collected during the second wave of the study (May 2013-2016). The collection of MCTQ was performed during a more limited period of the study (January 2016-November 2018). From the original sample, 597 volunteers met the criteria and were included in the analysis (age: 44.15 ± 13.73, ranging from 18 to 88 years old, 63.65% female). The sample was derived from 114 families with an average of five individuals from each family. Further details about the distribution of MEQ (von Schantz et al., 2015) and MCTQ (Ruiz et al., 2020) in samples from the same population have been presented previously. The distribution of the different measures in the current sample is shown in Supplemental Figure 1.

Chronotype was assessed by the following distinct instruments: The Brazilian Portuguese version of the MEQ (Benedito-Silva et al., 1990) is a scale containing 19 questions. Chronotype scores range from 16 to 86 points, with higher scores indicating morningness. The Brazilian Portuguese version of the MCTQ defines chronotype as a function of a phase of sleep and mid-sleep phase corrected for sleep debt accumulated over the working week (Roenneberg et al., 2003). Question 19 of the MEQ asks “One hears about ‘morning’ and ‘evening’ types of people. Which ONE of these types do you consider yourself to be?” The possible answers include definitely a “morning” type; rather more a “morning” type than an “evening” type; rather more an “evening” type than a “morning” type, and definitely an “evening” type.

We also scored from the answers to the MEQ the recently reported underlying factors (Panjeh et al., 2021). The “sensitivity to the build-up of sleep pressure” factor was calculated from the sum of the scores for Questions 2, 10, and 12, theoretically ranging from 2 to 14 points. The “efficiency of dissipation of sleep pressure” was calculated from the sum of the scores for Questions 1, 3, 4, 5, 7, 9, 13, and 19, theoretically ranging from 6 to 35 points. Higher scores for both variables are associated with increased morningness.

Descriptive statistics were used to both characterize the study sample and describe central tendency, dispersion, and distribution properties of chronotype and derived variables. Sex differences for each trait were examined with unpaired *t* tests. Pearson correlation was used to explore the associations between age and each trait, and Spearman correlation was used to describe associations between MEQ Question 19 score and mid-sleep, and dissipation and build-up of sleep pressure *p* values <0.05 were considered significant. All data processing, analysis, and visualization were performed using Python (v3.7).

Heritability was computed for each trait and was obtained through the maximum likelihood estimate–based variance components approach using the Sequential Oligogenic Linkage Analysis Routines (SOLAR Eclipse version 8.4.2) (Almasy and Blangero, 1998). The estimate of heritability (h^2^r) is the ratio of the variance of each trait explained by the additive polygenic effects to the total variance for the individual measure. Estimates of the mean and variance components were obtained using maximum likelihood methods (Almasy and Blangero, 2010). For each trait, two models were fitted to the data: (1) unadjusted, which did not include any fixed effects, and (2) age and sex added as fixed effects. The best model refers to the final model where only significant covariates were kept as fixed effects. Data were obtained from 597 volunteers (63.6% women) aged 14-88 years (M age: 44.1, SD: 13.7) who completed both the MEQ and the MCTQ. All variables were within the normal range of distribution (kurtosis for MEQ total score = −0.0964, dissipation of sleep pressure = −0.4487, build-up of sleep pressure = −0.4543, MEQ Question 19 = −0.6059) with the exception to mid-sleep time on free days corrected for sleep debt on work days (MSFsc) (kurtosis = 2.7693), which was inverse-transformed as suggested by SOLAR and reached normalcy parameters after transformation (kurtosis = −0.0386). Measures are summarized in Table 2. Apart from the “dissipation of sleep pressure” factor, women and men did not differ in terms of the studied phenotypes. Moreover, the build-up of sleep pressure was the only trait that was not sensitive to age.

**Table 2.** Summary of collected measures, adjusted for sex and age.

Variable	M ± SD	Association With Sex	Association With Age
MEQ score	62.8 ± 9.9	ns	*r* = 0.43
MSFsc (mid-sleep time on free days corrected for sleep debt on work days), min past midnight	193 ± 79	ns	*r* = 0.34
MEQ Question 19 score “Definitely a morning type” = 6 points, “Rather more a morning type” = 4 point, “Rather more an evening type than a morning type” = 2 points, “Definitely an evening type” = 1 point	4.0 ± 1.9	ns	*r* = 0.29
Dissipation of sleep pressure (MEQ factor, scale points)	26.3 ± 5.7	Unpaired *t* test, *t* = 2.89, *p* < 0.05*	*r* = 0.46
Build-up sleep pressure (MEQ factor, scale points)	9.9 ± 2.5	ns	ns

Abbreviation: MEQ = morningness-eveningness questionnaire.

*Note*: *p < 0.05

Significant heritability was observed in the adjusted models for all measures of chronotype, the two established questionnaires, and the single question, as well as the MEQ factor analysis constructs (Table 3). The significant effect of age and sex on the heritability computation of MEQ corroborates the already well-established roles of these covariates. Equally, the observation of a significant effect of age but not for sex is consistent with the reports that sex differences in MCTQ are no longer present from middle age onwards (Roenneberg et al., 2004).

**Table 3.** Heritability (h^2^) estimates for the trait explored in this study.

Trait	Model	h^2^ ± SE	*p* Value	*p* Value of Covariates	Variance Explained by Final Covariates
MEQ score	Unadjusted	0.19 ± 0.10	0.0295		
	Adjusted best model	0.37 ± 0.11	0.0001	Age (5.63 × 10^−31^) Sex (0.0464)	0.1943
MSFsc, min past midnight	Unadjusted	0.17 ± 0.09	0.0295		
	Adjusted best model	0.32 ± 0.10	0.0002	Age (8.75 × 10^−20^)	0.1258
Dissipation of sleep pressure (MEQ factor, scale points)	Unadjusted	0.15 ± 0.10	0.0587		
	Adjusted best model	0.32 ± 0.10	0.0004	Age (1.32 × 10^−34^) Sex (0.0001)	0.2256
Build-up sleep pressure (MEQ factor, scale points)	Unadjusted	0.26 ± 0.11	0.0046		
	Adjusted best model	0.28 ± 0.11	0.0025	Sex (0.0362)	0.0055
MEQ Question 19 score	Unadjusted	0.17 ± 0.11	0.0498		
	Adjusted best model	0.28 ± 0.11	0.0043	Age (4.34 × 10^−14^)	0.0850

Abbreviations: SE = standard error; MEQ = morningness-eveningness questionnaire; MSFsc = mid-sleep time on free days corrected for sleep debt on work days. Unadjusted and adjusted models for each trait, as well as the significant covariates, are presented.

The parameter that showed the strongest correlation with MEQ Question 19 score was dissipation of sleep pressure (*r* = 0.718), followed by total MEQ score (*r* = 0.703), MSFsc (*r* = −0.349), and build-up of sleep pressure (*r* = 0.259). Measures are shown in Table 4.

**Table 4.** Correlations between MEQ Question 19 score and MEQ total score, MSFsc, build-up of sleep pressure, and dissipation of sleep pressure (factors derived from MEQ).

	*r*	*p* value
MEQ, full questionnaire	0.703	5.47 × 10^−90^
MSFsc	−0.349	1.38 × 10^−18^
Build-up of sleep pressure factor	0.259	1.28 × 10^−10^
Dissipation of sleep pressure factor	0.718	1.43 × 10^−95^

Abbreviations: MEQ = morningness-eveningness questionnaire; MSFsc = mid-sleep time on free days corrected for sleep debt on work days.

MEQ, the oldest measure of chronotype, has a documented relatively high heritability, as might be expected from the fact that its questions pertain to intrinsic diurnal preference. The lower value (0.37) obtained here than in our previous study of another sample from the same cohort (0.480; von Schantz et al., 2015) probably reflects the smaller sample size in this study. A number of candidate gene associations with MEQ have been reported, and at least some of them have been reproduced in different samples. By contrast, there are no studies quantifying the heritability of MCTQ and there are no reported genetic associations. As the component questions pertain to actual rather than preferred timings, it has been an open question whether MCTQ measures a state rather than a trait (Roenneberg et al., 2019). The fact that heritability estimates for both scales were derived from the same group of individuals allows us to conclude that despite being somewhat lower than that of MEQ, the genetic variance in MCTQ was significant. This is important because MCTQ data have been collected worldwide at a large scale (Roenneberg et al., 2019). Our findings suggest that the MCTQ score has a significant genetic dimension, which is resistant to social pressures on sleep and wake time, and therefore, has potential for genetic analysis.

Circadian signals and the kinetics of sleep pressure—both the build-up and the dissipation—differ across the chronotype continuum. As a result, evening types have a slower build-up and dissipation of the homeostatic sleep process in real-life conditions (Taillard et al., 2003). Based on EFA, Panjeh et al. (2021) unveiled the underlying latent factors of the MEQ linked to the homeostatic control of sleep. Our results demonstrated that both the MEQ factors described as “dissipation of sleep pressure” and “sensitivity to the build-up of sleep pressure” have heritability values lower than the MEQ, but similar to MCTQ. This supports the role of genetic regulation on the oscillatory sleep-wake patterns determined by the two endogenous processes (Dijk and Archer, 2010).

Manifestly, the use of a single chronotype question in large populations has been very successful in GWAS analyses both in terms of the number of associations identified and their levels of statistical significance. To a larger extent than for other behavioral traits, this hypothesis-free method has independently identified associations with previously known candidate genes from molecular components of the circadian clock (von Schantz et al., 2021). Our findings suggest the former, with the single question and full MEQ showing phenotypic correlation in our dataset (*r* = 0.7) and a heritability for the single question of approximately three fourths of the one estimated from MEQ. This suggests that the single question captures a substantial part of the genetic variance in chronotype. This is reassuring, given how much easier it is to collect answers to a single question from large cohort populations, and consistent with the previous observation that the answer to this question shows an 89% correlation to the typological classification of the full MEQ (Adan and Almirall, 1991).

In summary, this first comparison of the heritability of the major chronotype instruments in current use provides the first quantification of the genetic component of the MCTQ score, suggesting that it could be successfully used for future genetic analyses. Our findings also suggest that the single chronotype question that has been used for large GWAS analyses captures a larger proportion of the dimensions of chronotype than previously reported and assumed.

## Supplemental Material

sj-pdf-1-jbr-10.1177_07487304211030420 – Supplemental material for Compared Heritability of Chronotype Instruments in a Single Population Sample Supplemental material, sj-pdf-1-jbr-10.1177_07487304211030420 for Compared Heritability of Chronotype Instruments in a Single Population Sample by Mario A. Leocadio-Miguel, Francieli S. Ruiz, Sabrina S. Ahmed, Tâmara P. Taporoski, Andréa R. V. R. Horimoto, Felipe Beijamini, Mario Pedrazzoli, Kristen L. Knutson, Alexandre C. Pereira and Malcolm von Schantz in Journal of Biological Rhythms
