# Supplementary figures and images for: Compared Heritability of Chronotype Instruments in a Single Population Sample
Source: J Biol Rhythms. 2021 Jul 27;36(5):483–90. doi: 10.1177/07487304211030420 (PMC8442136; doi:10.1177/07487304211030420)

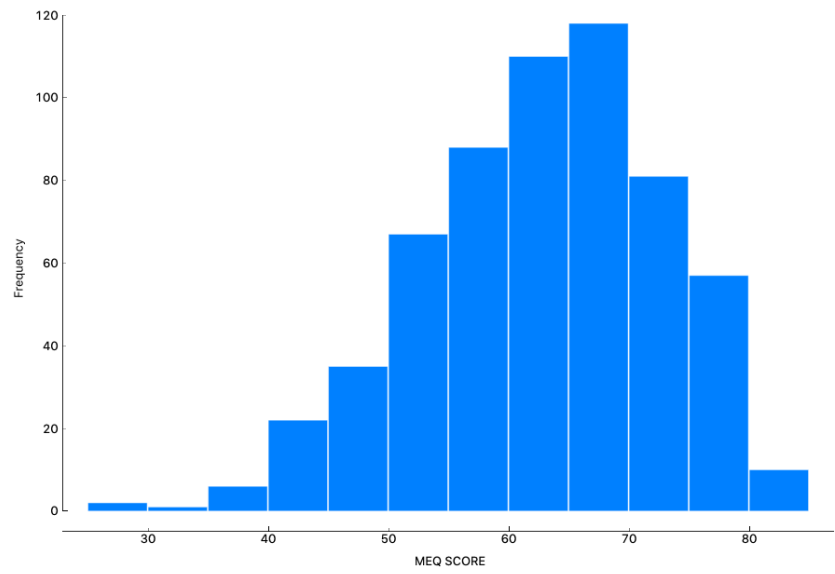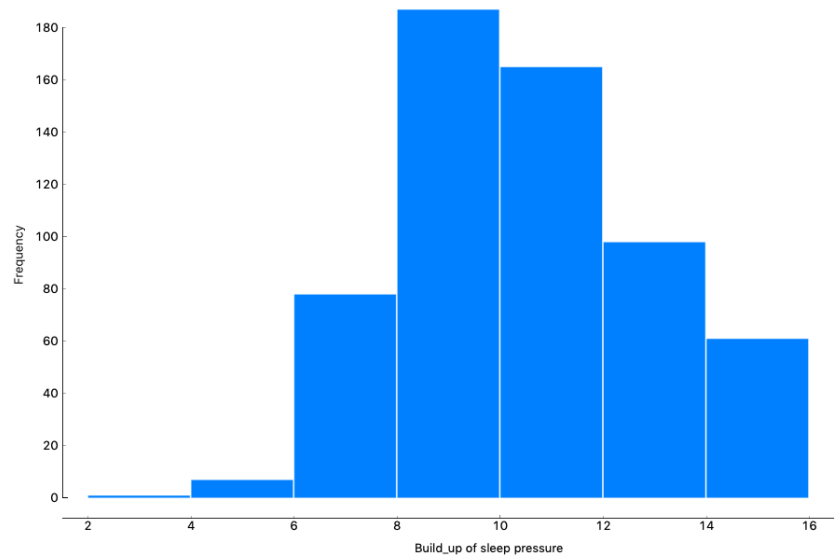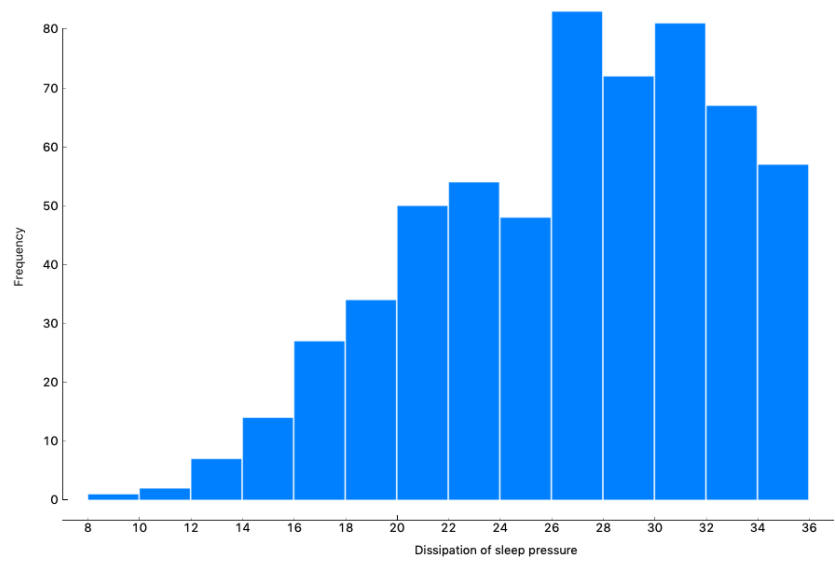

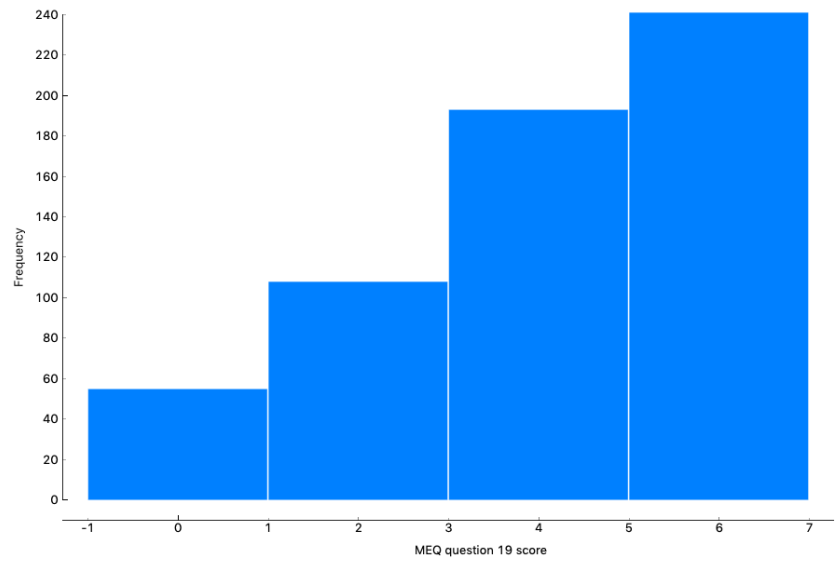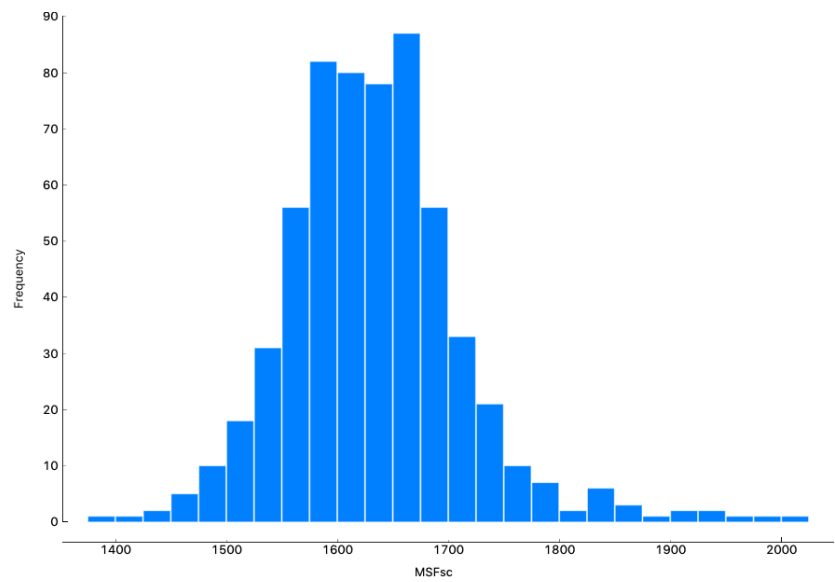

Supplement: sj-pdf-1-jbr-10.1177_07487304211030420 – Supplemental material for Compared Heritability of Chronotype Instruments in a Single Population Sample [file sj-pdf-1-jbr-10.1177_07487304211030420.pdf]
